# Nutritional Approaches for the Management of Nonalcoholic Fatty Liver Disease: An Evidence-Based Review

**DOI:** 10.3390/nu12123860

**Published:** 2020-12-17

**Authors:** Marcela Parra-Vargas, Roberto Rodriguez-Echevarria, Josep C. Jimenez-Chillaron

**Affiliations:** 1 Endocrinology Division, Institut de Recerca Sant Joan de Déu, Esplugues de Llobregat, 08950 Barcelona, Spain; marcela.parrav@gmail.com; 2 Hospital Sant Joan de Déu, Esplugues de Llobregat, 08950 Barcelona, Spain; 3 Institute of Translational Nutrigenetics and Nutrigenomics, Department of Molecular Biology and Genomics, CUCS, University of Guadalajara, Guadalajara, Jalisco 44340, Mexico; roberto.rodriguez@academico.udg.mx

**Keywords:** DASH diet, dietary therapy, intermittent fasting, ketogenic diet, low-carbohydrate diet, low-fat diet, Mediterranean diet, nonalcoholic fatty liver disease, nutritional management, obesity

## Abstract

Nonalcoholic fatty liver disease (NAFLD) is on the rise worldwide representing a public health issue. Its coexistence with obesity and other metabolic alterations is highly frequent. Therefore, current therapy interventions for NAFLD are mainly focused on progressive weight loss through modulation of overall calorie intake with or without specific macronutrient adjustments. Furthermore, other relevant nutritional interventions are built on food selection and time-restricted eating. Since every strategy might bring different results, choosing the optimal diet therapy for a patient is a complicated task, because NAFLD is a multifactorial complex disease. Importantly, some factors need to be considered, such as nutrition-based evidence in terms of hepatic morphophysiological improvements as well as adherence of the patient to the meal plan and adaptability in their cultural context. Thus, the purpose of this review is to explore and compare the subtleties and nuances of the most relevant clinical practice guidelines and the nutritional approaches for the management of NAFLD with a special attention to tangible outcomes and long-term adherence.

## 1. Introduction

The worldwide prevalence of nonalcoholic fatty liver disease (NAFLD) has increased along with that of obesity over the last decades. In fact, the global prevalence of NAFLD is around 25%, and it has become the most prevalent chronic liver disease in Western countries due to its strong association with obesity [1,2]. The rise in the prevalence of these conditions seems to be largely explained by the exposure to an “obesogenic” environment. This complex and multidimensional scenario is composed by diverse factors that promote an individuals’ overall energy imbalance (i.e., towards a sustained positive energy balance) such as increased availability (food supply) and overconsumption of low-nutrient, energy-dense foods, the modern sedentary lifestyle, among others, leading a state of excess adiposity [3,4]. Obesity represents the centerpiece in the development of several metabolic complications such as insulin resistance, diabetes mellitus type 2 (T2DM), cardiovascular disease (CVD), and NAFLD [2,5]. The latter is a multifactorial metabolic disorder in which excessive intrahepatic fat accumulation is the hallmark feature. Occasionally, liver fat is accompanied by inflammation that causes more drastic morphological changes in the liver tissue [6]. It is important to remark that besides over-nutrition, certain types of undernutrition paradoxically may promote the development of fatty liver [7].

NAFLD encompasses a wide spectrum of liver damage that is categorized by histological examination. The least advanced stage of disease, simple steatosis (SS), is characterized by steatosis alone (defined as >5% hepatocytes containing lipid vesicles). Nonalcoholic steatohepatitis (NASH), which represents a more severe form of NAFLD, is defined by the presence of marked inflammation and hepatocyte ballooning with or without fibrosis [6]. NAFLD is a progressive disease, in which chronic hepatic inflammation is involved in the evolution of NASH to cirrhosis that represents a risk factor for the development of hepatocarcinoma [8].

Healthy lifestyle modifications, namely diet and physical activity, are the mainstay of the NAFLD therapy [9,10,11]. NAFLD is part of a complex network of metabolic disruptions in multiple tissues commonly associated with obesity [12], which consequently makes diet therapy a difficult endeavor. The present tendency of nutritional intervention leans towards on correcting unhealthy dietary factors that promote disease progression. Currently, the optimal nutritional management remains controversial, although there is general consensus that gradual body weight loss is the recommended standard of care for the treatment of NAFLD. Dietary energy restriction is a key element to achieve weight reduction, but its compliance depends largely of self-control, and consequently, diet adherence might become quite challenging in most cases. Hence, different choices of nutritional interventions have been explored in the NAFLD context. This review is meant to serve as a comprehensive overview of the most relevant literature describing nutritional/dietary approaches for the management of NAFLD.

## 2. Methods

We searched the MEDLINE (https://pubmed.ncbi.nlm.nih.gov) database for relevant data in humans including clinical guidelines, clinical trials, reviews, and meta-analyses published from 2005 to 2020. In the search strategy, we used the keywords: “NAFLD” or “fatty liver” and “clinical guidelines” or “diet” or “management” for a first screening. Relevant diet therapies were detected from this first view. Then, a second search round included the search terms: “NAFLD” or “fatty liver” and “low fat diet” or “low carbohydrate diet” or “carbohydrate restricted diet” or “fat restricted diet” or “very low carbohydrate diet” or “ketogenic diet” or “dietary approaches to stop hypertension” or “DASH” or “Mediterranean diet” or “intermittent fasting”. In addition, we screened the citation list of the selected articles to obtain additional references. Relevant trials and meta-analyses conducted on adult and pediatric population that evaluated dietary effects preferably on intrahepatic fat content, and/or other histological features along with body weight were included. Studies evaluating the dietary effect coupled with other nutritional intervention (e.g., antioxidant supplementation) were excluded. This review is neither a systematic review nor meta-analysis.

## 3. Nutritional Management of NAFLD

### 3.1. Clinical Practice Guidelines

Clinical practice evidence-based guidelines establish that lifestyle modifications are fundamental in the treatment of NAFLD. Essentially, dietary energy restriction and regular physical activity are prescribed to promote body weight reduction and improve hepatic steatosis as well as other histological features of NAFLD [9,10,11]. Table 1 summarizes the overall nutritional/lifestyle interventions suggested by the most recent international guidelines for NAFLD management. According to these guidelines, gradual body weight loss is a primary target in subjects with overweight and obesity. In particular, the American Association for the Study of Liver Diseases (AASLD) recommends a total weight reduction of at least 3–5% to ameliorate steatosis, whereas improving most of the histopathological features requires a greater degree of weight loss (7–10%) [10,11]. The European Association for the Study of the Liver (EASL), European Association for the Study of Diabetes (EASD), and European Association for the Study of Obesity (EASO) suggest a 7–10% of total weight loss target [9]. The EASL-EASD-EASO also advises the use of structured programs aimed at lifestyle changes.

### 3.2. Dietary Strategies in the Nutritional Management of NAFLD

Dietary patterns and nutrients play a key role in the development, progression, and treatment of NAFLD and its metabolic-associated comorbidities. The onset and progression of NAFLD is tightly associated with an unhealthy dietary pattern. Several studies have shown that there is a common eating pattern in NAFLD patients, which is characterized by low intake of whole grains, cereals, fruits, and vegetables, whereas the intake of red meat, viscera, refined-grains, and/or sugars are higher than the recommended amounts [13,14,15]. These eating habits are typically found in the Western pattern diet, which is highly related with the development of multiple metabolic diseases including NAFLD [13,14,15]. A recent meta-analysis indicated that consumption of red meat and soft drinks is related with an increased likelihood of NAFLD [16]. Likewise, high intake of red meat is associated with insulin resistance [15]. In fact, the Western diet is usually described as being high in total energy with an elevated content of saturated fat and refined sugars. These macronutrients influence metabolic pathways that contribute to liver fat accumulation. For example, a hyperenergetic diet with excess energy predominantly from saturated fatty acids elevated intrahepatic triglycerides (55%) by stimulating adipose tissue lipolysis. On the other hand, extra energy from simple sugars increased liver triglycerides (33%) by stimulating de novo lipogenesis [17]. Remarkably, we wish to stress out that although saturated fats have been under the spotlight for decades as a factor promoting CVD, there is growing evidence showing that this classification might be strongly biased [18]. Together, these studies support that nutrition is a major factor influencing disease risk, and therefore, diet is a key modifiable risk factor in NAFLD treatment.

A network meta-analysis that included 19 randomized-controlled trials reported that exercise plus diet is the most effective intervention for NAFLD management [19]. Lifestyle intervention in NAFLD is mainly aimed to reduce body weight due to its strong correlation with improvements in intrahepatic fat among other parameters. In a randomized clinical trial, the degree of weight loss due to a lifestyle intervention predicted NAFLD remission in both patients with and without obesity. Importantly, the amount of weight reduction required to achieve the remission was lower in non-obese cases (3–10%) compared to those with obesity (7–10%) [20]. Histologically, improvements in steatosis, liver inflammation, ballooning degeneration, and fibrosis have also been observed in NASH when weight loss was higher (≥7–20%) [21,22,23]. In principle, energy restriction is the most effective dieting method for weight loss. However, long-term weight loss and maintenance by limiting energy is quite challenging for most patients, which curtails long-term outcomes. Noteworthy, the number of meals per day has also been a focus of debate since >3 meals per day (adding snacks) produces more frequent insulin spikes, which in turn, might increase food craving throughout the day and consequently, it interferes with optimal diet adherence [24]. Hence, alternatively to cutting down on overall energy intake, multiple dietary strategies including low-fat diet (LFD), low-carbohydrate diet (LCD), ketogenic diet (KD), the Dietary Approaches to Stop Hypertension (DASH) dietary pattern, and the Mediterranean dietary pattern (MedDiet) have been evaluated in the NAFLD setting (Table 2 and Table 3 for review). In great measure, these dietary approaches consider not only quantitative but also qualitative nutritional features. Furthermore, alternatively to the traditional continuous energy-restricted diets, the intermittent fasting (IF) approach has become more popular for body weight management and it has received a special attention as a potential novel dietary therapy for NAFLD. Thus far, optimal nutrition and precise dietary management for NAFLD remains a matter of intense debate.

#### 3.2.1. Low-Fat or Low-Carbohydrate. Which Diet Is More Suitable for NAFLD Treatment?

The optimal macronutrient distribution to improve the clinical features related with NAFLD remains unclear. Nonetheless, based on the current literature, manipulation of the dietary macronutrient composition by simply varying the relation between total fat and carbohydrate content with or without energy intake restriction is advisable. Such dietary strategies are mainly designed to limit the intake of fat and/or carbohydrates with relatively minimal changes in that of the protein. Specifically, the LFD is an eating regimen in which the energy from fat sources is limited (Table 2). Commonly, a LFD restricts energy from fat to <30% of total daily kcal, while less than 20% is considered as a very low-fat diet [41]. On the other hand, the LCD is a regimen that restricts the overall intake of carbohydrates (Table 4). Although there is not a clear standard criterion on what defines a LCD at this time, restriction protocols range from 50 to 130 g/day, or 10 to <40% from daily energy [42]. Very low-carbohydrate diets provide even lower amounts of carbohydrate, i.e., <20 to 50 g/day, or <5 to 10% of daily kcal, which puts the body into ketosis state; and therefore, it is referred as KD [42,43]. The effects of KD in the NAFLD context are discussed below in an independent section.

Both LFD and LCD have been in and out of fashion for decades and have been broadly studied in regards of their effectiveness on body weight reduction. For several years, research had been focused on answering the basic long-standing question regarding if any of these two diets is a better dietary choice for body weight management. Based on the Diet Intervention Examining The Factors Interacting with Treatment Success (DIETFITS) randomized clinical trial, LFD and LCD interventions for 12 months were comparable in regards of weight loss (−5.3 for LFD vs. −6.0 kg for LCD) [44]. A meta-analysis of randomized clinical trials that included 53 studies found that long-term effect (≥1 year of follow-up) on weight loss was slightly greater with a LCD compared to LFD (weighed mean difference: 1.15 kg, 95% CI: 0.52 to 1.79) when the intensity of intervention was comparable [45]. It should be mentioned that LCD without restriction on total energy intake has equally proven to be an effective tool. In a two-year trial the mean weight loss was lower with a low-fat, restricted-energy diet compared to a low-carbohydrate, non-restricted-energy diet (−2.9 vs. −4.7 kg, respectively) [25]. Additionally, both low-fat and low-carbohydrate dietary regimens have proven similarly effective in ameliorating alterations associated with metabolic syndrome [46]. In brief, there is robust evidence to widely recommend either a LFD or LCD for weight management. Nevertheless, at this time it is still not clear which approach is clinically superior and most likely to provide sustained weight reduction and long-term beneficial metabolic outcomes. An important point to consider for LCD is that it has shown effectiveness for reducing body weight, HbA1c, serum lipids, and blood pressure in patients with T2DM and prediabetes after six years of treatment. Of course, these results show a clinical applicability in situations of chronic metabolic alterations [47].

With this in mind, the question is whether these dietary interventions are also effective for the treatment of NAFLD, which is part of the metabolic diseases associated to obesity and weight loss is highly advisable to improve NAFLD. A meta-analysis based on clinical trials reported that LCD, defined here as <50% of total energy from carbohydrates decreased intrahepatic lipid content by −11.53% (95% CI: −18.10, −4.96) in individuals with NAFLD [48]. Noteworthy, two studies included in this meta-analysis used KD and they will be discussed later in this article. There is much interest in identifying whether low-carbohydrate eating provides advantageous effects in the context of NAFLD compared to LFD. In a clinical trial, energy restriction by limiting dietary fat or carbohydrates for a 6-month period had comparable effect reducing body weight and intrahepatic lipids content (−42% vs. −47% for LFD and LCD, respectively) in individuals with overweight and obesity [26]. As both diets in this study were energy restricted in order to ensure body weight loss, the impact of such macronutrients on liver fat reduction is uncertain. However, there is evidence that modifying macronutrient distribution, even in the absence of limiting energy intake, the liver fat content is reduced. After 12-week ad libitum LFD, hepatic triglyceride content decreased by 25% even without a substantial change in energy intake from baseline and independently of body weight loss in adults with NAFLD [27]. In contrast to the previous study, a LFD in which energy was calculated to preserve body weight had no effect on hepatic fat after 8 weeks in adolescents with obesity and NAFLD. In the same study, despite prescribing the low-fat energy-balanced diet (based on the USDA guidelines for adolescents), only participants that underwent a LCD experienced weight loss (−0.4 vs. −2.4% for LFD and LCD, respectively) and hepatic fat declined by 32% [28]. Similar results were observed in an 18-month weight-loss trial conducted on adult participants with abdominal obesity/dyslipidemia. A low-carbohydrate MedDiet brought a greater decrease in hepatic fat content than LFD (−4.2% vs. −3.8% [absolute units], respectively) even after adjustment for visceral adipose tissue changes. This positive effect was significant even in patients without NAFLD as in those with the disease [29].

There is an ongoing debate on whether low-carbohydrate diet is more suitable than low-fat diets for treating NAFLD. A meta-analysis found that neither the low-carbohydrate (considering also very low-carbohydrate diets) nor low-fat diet is superior on ameliorating liver fat or transaminases levels in NAFLD. The authors of the meta-analysis suggest that more studies are warranted in order to address this issue [49]. Alternatively, it might also be possible that both types of diets have similar effects on promoting hepatic fat variation in NAFLD. Furthermore, beyond macronutrient quantity, there is evidence that the type and quality (food source) of the calories should be taken into consideration. Regarding carbohydrates, the quality of sources based on their postprandial glucose response may be a complementary useful dietary tool in the NAFLD management. Indeed, some studies including the low-glycemic scheme within their nutritional interventions have shown beneficial effects in the liver fat content [35,50,51].

#### 3.2.2. Very Low-Carbohydrate Diets: The Ketogenic Diet Therapy

Undoubtedly, the KD has favorable effects of body weight. Based on a meta-analysis, KD resulted in greater long-term reductions in body weight in comparison to a LFD [52]. In addition, consistent findings suggest a positive effect of KD on liver fat in short and medium term. A 6-day KD rapidly decreased intrahepatic triglycerides by 31% in subjects with overweight and obesity. Likewise, body weight and hepatic insulin resistance were reduced by 3% and 58%, respectively. In this recent clinical trial, the KD provided ∼1440 kcal/day in which the macronutrient distribution was ∼6:∼64:28% from daily energy of carbohydrates, fat, and protein, respectively. Mechanistically, hepatic fat reduction was mainly attributed to increased net hydrolysis of liver triglycerides, and partitioning of the separated fatty acids toward ketogenesis (+232%) [36]. In agreement with this finding, 2-week KD (∼1550 kcal/day with <20 g/day carbohydrate intake) reduced both liver triglycerides (by ∼55%) and body weight (−4.6 kg) in individuals with obesity and NAFLD. In the same study, KD was more effective at reducing hepatic triglycerides than a low-energy diet (−28%), despite similar effects on body weight loss [30]. Furthermore, a six-month KD (<20 g/day carbohydrate intake) resulted in liver histology improvements including steatosis, and inflammation, with a tendency to ameliorate fibrosis in five biopsy-proven NAFLD patients with obesity, which also achieved weight reduction (−12.8 kg) [37].

Taken together this body of evidence, severe carbohydrate restriction led favorable outcomes in patients with NAFLD. However, it is not possible to discern if these effects were secondary to weight loss and/or total energy changes rather than carbohydrate distribution. Based on this argument, a 2-week intervention study showed that a carbohydrate-restricted (<30 g/day carbohydrate intake) and high-protein diet under isoenergetic conditions decreased liver fat (−43.8%) in subjects with obesity and NAFLD. To note, participants consumed an average of 3115 kcal/day, and energy intake was increased whenever their weight decreased by more than 0.2 kg in two days to separate out the effect of weight loss. Still, a minimal weight loss was observed (−1.8%). Reduction in hepatic fat content was likely associated with decreased de novo lipogenesis, and increased fatty acid oxidation [38].

Although KD has the potential to provide remarkable outcomes in NAFLD, this dietary approach is essentially a high-fat dietary regimen, which severely restricts all carbohydrates. Thus, it might lack fiber-rich foods as well as water-soluble vitamin sources in situations when the diet plan does not take these elements in consideration. In other words, KD combined along with the permissive plant-based diet components might be a better strategy that those more prone to select animal sources of fat. Importantly, there may, however, be risks associated with following a KD for a prolonged interval of time. In fact, the major issue is to demonstrate that long-term ketosis and food restriction are not harmful, and whether diet compliance and benefits are sustainable. In this regard, greater LDL-cholesterol levels associated with a KD during weight loss has been previously reported [52]. However, lipid profile changes raised by KD may need a deeper analysis, considering also lipid sub-fractionations [53]. Therefore, large-scale, long-term intervention trials with comparative arms are needed to prove safety and efficacy of KD in NAFLD. Interestingly, although the evidence for current reports of long-term in NAFLD is scarce, a KD diet has been proven effective for T2DM treatment, which in fact shows positive effects in a context of metabolic alterations [54].

#### 3.2.3. DASH Dietary Pattern

This healthy eating pattern was originally proposed in the DASH study in the 1990s [55,56]. For decades, the DASH diet has proven to lower blood pressure, and therefore it has been used as a lifestyle approach for preventing and treating hypertension [56,57,58]. Essentially, it is a sodium-restricted diet (<2400 mg/day), low in saturated fat, and rich in protein, fiber, calcium, magnesium, potassium, zinc, and folate [58,59]. The DASH dietary pattern emphasizes the consumption of fruits, vegetables, low-fat dairy products, whole grains, poultry, fish, nuts, seeds, and legumes, whereas fats, red meat, sweets, and sugar-containing drinks is reduced [59]. Adherence to the DASH diet had a significant inverse dose-response association with all-cause, CVD, stroke, and cancer mortality [60]. It was also associated with lower incident of diabetes [61].

The relationship between DASH diet and NAFLD has recently gained attention. Evidence from observational studies support that DASH dietary pattern could play a preventive role. A case-control study showed an inverse relationship between adherence to a DASH-style diet and risk of NAFLD. Participants in the top quartile of DASH diet score, i.e., a scale based on food and nutrients emphasized or minimized in the DASH diet, had a 30% lower risk of NAFLD. However, after adjusting for main risk factors such as dyslipidemia and body mass index (BMI), the significance of this association disappeared [62]. Besides, a 20-year follow-up study found that higher quality diets during mid-to-late adulthood were associated with a lower risk of NAFLD in a multiethnic cohort subgroup with nearly 2000 participants. The risk decreased in the upper tertile of diet quality was significant for DASH among other dietary indices even after adjustment for total body fatness [63]. The nested case-controls analysis carried out within the multiethnic cohort that involved 2959 NAFLD cases (509 with cirrhosis and 2450 without cirrhosis) and 29,292 matched controls showed that higher DASH scores were inversely associated with NAFLD risk [64]. In agreement, the DASH score was related to lower liver fat content and NAFLD in a recent cross-sectional study [65].

With respect to interventional trials, the DASH dietary pattern has been linked with improvements in blood pressure, body weight, insulin homeostasis, and lipid profile [66,67]. A systematic review and meta-analysis of randomized controlled clinical trials reported that this eating pattern is a suitable option for weight loss and might be a better choice than low-energy diets [67]. However, its efficacy on NAFLD management is scarcely explored. A randomized controlled trial conducted for 8 weeks in patients with overweight/obesity and NAFLD showed significant beneficial effects on: (1) body weight, (2) liver transaminases, (3) markers of insulin sensitivity, (4) lipid profile, (5) serum biomarkers of inflammation and, (6) oxidative stress in the DASH diet group compared to low-energy diet [31]. The DASH diet pattern may be beneficial in NAFLD. Additional studies, in particular comparative clinical trials, are needed to investigate the impact of DASH diet on NAFLD features including histological and metabolic parameters.

#### 3.2.4. Mediterranean Dietary Pattern

The MedDiet is a nutritional approach inspired in the traditional eating pattern of countries located near to the Mediterranean Sea during 1960s [68]. In the Seven Countries Study, the typical eating habits of populations from the southern Europe were associated with lower rates of incidence, and mortality from coronary heart disease [68,69]. Since this first epidemiological study, more solid evidence associating MedDiet with a lower risk of CVD has been collected and the MedDiet definition has evolved and varied [70,71,72]. While there is no a unique definition of MedDiet, this cultural model for eating is essentially characterized by high intake of plant-derived foods and limited consumption of refined sugars and processed foods. The traditional MedDiet comprises nine characteristics: (1) high consumption of olive oil, which is the primary fat source, and the use of extra-virgin olive oil is abundant, (2) legumes, (3) cereals as whole grains, (4) fruits and (5) vegetables, (6) moderate to high consumption of fish, (7) low consumption of meat and meat products, and (8) moderate consumption of dairy products, mostly as cheese and yogurt, and (9) wine [73,74]. In general, MedDiet provides around 13–18% of energy from protein, 43–48% from carbohydrates, and 35–41% from fat, and it tends to be low in saturated fat whereas monounsaturated fatty acids (MUFA) intake is especially abundant (8–11% from saturated fat, 16–23% from MUFA, 4–6% from polyunsaturated fats). Furthermore, it provides approximately 33 g of fiber per day [70].

The MedDiet has been recognized worldwide as a healthy dietary pattern. A large body of research supports the effectiveness of MedDiet in major chronic disease prevention and management [71,72,73,75,76]. Over the past several years, the relationship between MedDiet and NAFLD has received special attention. A recent systematic review and meta-analysis from observational and clinical investigations have shown a trend for the relationship between MedDiet and hepatic steatosis. Importantly, improvement in BMI, plasma triglycerides, and insulin resistance (HOMA-IR) may be involved in such relationship [77]. Indeed, available knowledge from observational studies supports the notion that the MedDiet is a dietary pattern able to prevent NAFLD. In a cross-sectional study (*n* = 73), a greater adherence to MedDiet, estimated by MedDiet score, correlated with less severe liver steatosis, and lower degree of insulin resistance in patients with NAFLD. However, greater adherence to MedDiet was not associated with a lower probability of having NAFLD when subjects were compared with age, sex, and BMI matched control group [78]. In line with these findings, another cross-sectional study conducted on individuals (*n* = 584) presenting cardio-metabolic risk factors (i.e., T2DM, arterial hypertension, overweight/obesity, and dyslipidemia) reported an inverse relationship between MedDiet and NAFLD prevalence. Furthermore, good adherence to MedDiet correlated with lower insulin resistance among patients with NAFLD [79]. In a recent a cross-sectional analysis of two population-based adult cohorts (the Fenland Study, England, *n* = 9645; and CoLaus Study, Switzerland, *n* = 3957), greater adherence to the MedDiet correlated with lower prevalence of hepatic steatosis, and this finding was largely explained by adiposity [80]. Besides, in pediatric population with obesity, a low adherence of MedDiet (evaluated by a clinical questionnaire, the Mediterranean diet quality, index for children and adolescents, KIDMED) was significantly higher in NASH cases, and poor adherence correlated with liver damage (NAFLD activity >5, and grade 2 fibrosis), and higher fasting insulin levels [81]. 

Over the last few years, interventional studies have demonstrated favorable effects of the MedDiet on NAFLD. In a 2-year randomized clinical trial a moderate-fat, restricted-energy, MedDiet in subjects with obesity resulted in greater body weight reduction and lower alanine aminotransferase (ALT) levels compared to low-fat restricted-energy diet. Moreover, MedDiet provided some additional metabolic benefits, especially on glycemic control among participants with diabetes [25]. Furthermore, MedDiet has shown to reduce hepatic steatosis. A pilot crossover trial reported that MedDiet for 6 weeks significantly reduced hepatic steatosis (−39%) compared to subjects that undertook a low fat-high carbohydrate diet (−7%), and it also improved insulin sensitivity even without weight loss in individuals with insulin resistance and NAFLD [32]. However, in a single-arm study focused to increase adherence to MedDiet and physical activity in patients with NAFLD, reductions in hepatic fat content were significant only after 6 months intervention. Interestingly, variance of the liver fat decrease was explained by changes in the adherence to MedDiet and BMI, independently of other lifestyle changes [39]. The long-term effects of an energy-unrestricted MedDiet supplemented with either extra virgin olive oil or nuts on diverse conditions have been compared with a LFD in the Spanish multi-center, randomized “Prevención con Dieta Mediterránea” (PREDIMED) study conducted on individuals with high cardiovascular risk. Within the frame of this study, data from a 6-year follow-up analysis suggested that MedDiet could delay or slow down the progression of NAFLD [33]. Besides, according to a 3-year follow-up analysis, MedDiet rich in extra virgin olive oil was associated with a reduced prevalence of NAFLD [34], suggesting a protective role of extra virgin olive oil against hepatic steatosis. In addition, a recent randomized controlled trial has compared the beneficial effects of two personalized healthy dietary approaches in the context of energy restriction in subjects with overweight/obesity and NAFLD. Results showed that after a 6-month intervention period, both diets improved metabolic and hepatic markers in similar degrees. Interestingly, higher adherence to the MedDiet along with weight loss predicted up to 40.9% variability in liver fat content after 6 months [82].

Some studies have examined the efficacy of the Mediterranean eating pattern along with changes in nutrient distribution and/or considering quality features. In this context, the effects of a Spanish ketogenic MedDiet were evaluated in a pilot study carried out in subjects with metabolic syndrome and NAFLD. Participants undertook for 12 weeks an energy-unlimited KD (<30 g/day carbohydrate intake) combined with four essentials components of the MedDiet: (1) virgin olive oil, and ω-3 fatty acids from fish as the main fat sources, (2) fish as main protein source, (3) green vegetables and salads as main carbohydrate source, and (4) moderate red wine consumption. Results showed that body weight and steatosis degree decreased compared to the basal values among other metabolic parameters. Indeed, this diet induced a complete fatty liver regression in 21.4% of patients with a 92.86% of overall reduction. Levels of ALT and aspartate aminotransferase (AST) also decreased [40]. Similar results were observed in the previously mentioned study using a low-carbohydrate MedDiet for 18 months, in which intervention decreased hepatic fat content in a greater manner than a LFD [29]. The effect of a low-glycemic index MedDiet in individuals with moderate or severe NAFLD was assessed in a 6-months randomized clinical trial. In this study, the energy-unrestricted MedDiet that contained all foods with low-glycemic index was compared with a healthy diet. A greater decrease in the NAFLD score was observed with the low-glycemic MedDiet. Moreover, it was reported a negative interaction between time and MedDiet on the NAFLD score [35].

According to the AASLD, a minimal body weight loss (3–5%) is necessary to improve steatosis, and it is the most important factor in NAFLD treatment [10,11]. However, as weight loss is indeed an outcome of a lifestyle intervention, eating habits might have a greater impact on remission of NAFLD rather than weight loss per se. In this sense, the MedDiet is a high-quality dietary pattern-based approach that represents a clinically feasible alternative in the NAFLD management. Some evidence has shown favorable changes in metabolic parameters and hepatic fat content even with a slight weight reduction [27,32,39]. In fact, an ad libitum MedDiet for 12 weeks decreased hepatic triglyceride content (−32.4%), liver enzymes levels with a minimal weight loss (−2.1 kg) in patients with NAFLD, and there was no significant difference compared with LFD [27]. Hence, eating habits based on high-quality food choices may play an important role in the NAFLD treatment independent of limiting the energy intake and weight reduction. Accordingly, the EASL-EASD-EASO Clinical Practice Guidelines and the European Society for Clinical Nutrition and Metabolism (ESPEN) guidelines advise the MedDiet for treating the disease [9,83]. The transferability of the MedDiet to non-Mediterranean populations is feasible, although it could be a quite challenge or no practical particularly in those populations with deep food cultural differences. A few studies have been carried out in non-Mediterranean populations [25,27,29,32]. In this regard, a randomized controlled trial of a Mediterranean dietary intervention for adults with NAFLD (MEDINA) is using a MedDiet model intervention in non-Mediterranean population. This study is currently underway and is aimed to demonstrate that 12-week MedDiet can result in significant benefits in liver fat and insulin sensitivity and these changes are sustained at 12 months in patients with NAFLD who are insulin resistant. This dietary model incorporated key elements of MedDiet, and preserved traditional dietary components even with the adaptation to an Australian multiethnic population [84]. In this context, careful attention should be paid to the traditional MedDiet features aforementioned. A substantial reduction (or even total annulment) of these elements differs to the concept of the traditional MedDiet, and these should be preserved to obtain this transferability and ensure the reported health benefits [74].

In addition to the dietary component, the Mediterranean lifestyle also involves regular physical activity, and healthy sleep habits. In this regard, a recent randomized controlled single-blinded clinical trial, evaluated the effects of a MedDiet and a Mediterranean lifestyle (moderate-vigorous intensity, at least 30 min/day; optimal sleep duration: ≥7 and ≤9 h/day, and mid-day rest) compared with usual care in patients with NAFLD. At the end of 6-month follow-up period, the Mediterranean lifestyle improved ALT levels and liver stiffness, while the MedDiet only improved liver stiffness compared to the control group (after adjusting for percentage of weight loss and baseline values). This evidence suggests that small modifications towards the Mediterranean lifestyle, along with weight loss could be a successful treatment option in NAFLD [85]. The MedDiet is a helpful dietary strategy in the NAFLD management. It should be pointed out that the MedDiet efficacy relies on the whole diet rather than on its individual components. More evidence demonstrating the impact of the MedDiet on other NALFD features such as hepatic inflammation and fibrosis is required. Likewise, further research on the transferability and adherence to the MedDiet in non-Mediterranean populations particularly in those with high NAFLD prevalence is needed.

#### 3.2.5. Intermittent Fasting: The Non-Continue Energy Restriction

This diet strategy implies an automatic energy intake reduction by restricting the feeding period. The term IF encompasses various types of energy restriction on an intermittent basis that consists in alternating periods of eating and fasting with either total food abstinence or very low energy intake, on a regular schedule [86]. The most common IF methods are alternate-day fasting (ADF), time-restricted feeding (TRF), and 5:2 diet [86]. The IF has gained popularity as an approach for weight management. In fact, a systematic review and meta-analysis that included six interventional trials concluded that IF had comparable effects with continuous energy restriction for short-term weight loss in adults with overweight and obesity [87]. As the primary therapy goal in NAFLD is weight loss, it is expected that the IF will positively influence liver steatosis and related metabolic alterations. Therefore, the intermittent energy restriction by fasting approach is a promising therapeutic method for NAFLD.

An observational cohort study (*n* = 697) was conducted to investigate the effects and safety of periodic fasting in individuals with and without diabetes. Data from this trial showed that after a mean period of 8.5 days of fasting (a maximum of 250 kcal/day energy intake), 120 of the 264 individuals with fatty liver index ≥60 (i.e., in the highest risk category of fatty liver) changed to lower risk category. Importantly, a significantly greater reduction was observed in subjects with diabetes than non-diabetic cases (−19.15 vs. −13.73 points, respectively). Besides, the improvement in the fatty liver index score correlated with the duration of the fasting and the magnitude of weight loss and BMI reduction (−5%) [88]. Although periodic fasting was well tolerated in this study, fasting for many days to achieve long-term effects can be challenging and incompatible with social life, where eating is indeed a social activity. Recently, one randomized trial compared ADF (altering 24 h fast with a 25% of daily energy supply and 24 h feeding ad libitum) and TRF (8 h feeding and 16 h fasting) with a control group (diet providing 80% of energy needs) for 12 weeks in patients with NAFLD. Results revealed that both fasting interventions induced a significant weight loss in a short period compared to control (ADF: −4.04 kg, TRF: −3.25 kg vs. control: −1.17 kg). However, no changes were observed in insulin resistance and liver stiffness among other parameters [89]. Another study reported that a modified alternate-day energy restriction (alternating a fasting day with a restriction of 70% of energy requirement and non-fasting day ad libitum) for 8 weeks led to a significant reduction of liver steatosis and fibrosis grades, BMI, and ALT compared to an usual habitual diet (i.e., no intervention) [90].

IF is common practice in some religious cultures (e.g., Islamic Ramadan) [91]. Therefore, religiously motivated fasts are a great opportunity to study health-related effects of fasting. In an observational trial (*n* = 83), Ramadan fasting (15–16 h/day during a month) significantly decreased body weight and BMI among other anthropometric parameters in subjects with NAFLD, but not the control group (subjects with NAFLD who decided not fast). Furthermore, fasting improved metabolic parameters including glucose levels, insulin, and insulin resistance (HOMA-IR) [92]. In addition, the same research group published later that Ramadan fasting improved also liver enzymes and severity of hepatic steatosis [93]. Thus far, scientific evidence in humans is very limited. Notably, many questions have yet to be answered and the evidence gap needs to be filled before prescribing this dietary strategy to patients with NAFLD. Hence, long-term follow-up studies with comparative arms using magnetic resonance spectrometry or biopsy for detecting liver improvements are needed to establish efficacious and safety of the IF therapy in NAFLD. The IF could be challenging, but this type of energy restriction approach may result to be more self-control sustainable and practical than continuous energy restriction. Therefore, it is necessary to state the timing of meals and the minimum amount of fasting time to observe benefits in liver fat content, histological injuries, and other metabolic alterations as well as the timing of the fasts.

## 4. Future Perspectives in the Nutritional Management of NAFLD

NAFLD is a growing global public health issue that affects adult and pediatric populations and it is expected to worsen in the upcoming years due to its close association with the obesity epidemic and other obesity-related metabolic unhealthy phenotypes. Since nutritional therapy is fundamental in NAFLD management, it should be oriented to prescribe individualized high quality healthy diets instead of aiming at weight loss per se. Indeed, NAFLD management has long been focused on weight reduction mainly through low-energy dietary intervention with or without modified macronutrient distribution. This, in fact, is intended to gradually decrease low-grade chronic inflammation given that higher adiposity can be pinpointed as a major component of metabolic disorders. However, long-term adherence to low-energy diets is still a troubling issue due to two reasons: first, there is usually little attention or no further guidance on how to sustain the achieved benefits for an individual’s health over the years. Secondly, sustaining a high level of adherence in diets including alternative macronutrient distribution over months or even for years is virtually impossible for most individuals. Besides, promoting a reduced intake of one macronutrient for a long period of time might lead to other nutrient deficiencies (e.g., fiber intake could be compromised in carbohydrate-restricted diets as well as some vitamins contained in whole grains and green leaves such as thiamine, riboflavin, niacin and folate) that might worsen digestive or even metabolic functions if these are overlooked. Thus, it is advisable that the nutritional guidance from nutritionists includes foods with high fiber-net carbohydrates ratio (e.g., avocado, nuts, as well as chia seeds and flaxseeds).

Alternatively to dietary restriction, certain dietary patterns have shown promising results with a high degree of clinical relevance. These patterns are based on food selection considering high quality foods. Indeed, nutrient replacement founded on whole grain choices instead sugary foods or drinks have long been part of certain cultures, such as the MedDiet. This dietary pattern, for instance, has been widely acknowledged for the doubtless cardiovascular benefits it provides. However, not all countries would be able to initiate it, let alone adhere to a dietary pattern that might not share anything in common with local food production and traditional cuisine, which are deeply rooted into the history of different human cultures. Of course, it is not an availability issue what we wish to stress out here, but rather a cultural barrier that may represent a great challenge for many geographically distant populations when trying to adapt to different food choices.

Taken together the previous considerations, the “one-size-fits-all” idea is unable to be replicable on this matter as it has been in many other clinical nutritional areas. Therefore, it is important that the upcoming dietary clinical guidelines for NAFLD takes the cultural background into consideration before pushing for straightforward dietary interventions. In fact, a local and sustainable diet will always be preferred over new proposals because of the psychological, social, and economic positive impact it produces. Finally, a large number of foods, regardless of their origin, may be a source of antioxidants and other phytonutrients that enhance or inhibit certain cellular processes. In fact, there is a pipeline of understudied foods and food components that might do the job as well when prescribing strategic dietary plans. Thus, we are convinced that the health-regaining process through diet modification in NAFLD management should play more attention on the local sources of high-quality foods and a bit less in the calorie count.

## 5. Concluding Remarks

NAFLD prevalence is growing alongside that of obesity and it is likely to be so over the next decades. The most effective type of long-term treatment remains diet therapy. However, deciding on which diet regimen is best for a patient or group of patients is still a hard task due to the multiple factors that need to be considered. Among the most important features of the most popular diets in NAFLD we are able to list the following: overall calorie count, food choices, distribution of energy from fat and carbohydrates, and timing. Importantly, more research is needed to address the positive and possibly, the negative effects of the combinations of these approaches. Although these diets might produce different metabolic and hormonal effects separately or combined, we conclude that for long-term sustained health benefits, food choice should be a major priority. In other words, choosing local high-quality foods (e.g., whole grains, seeds, fresh fruits and vegetables as well as sources of monounsaturated fatty acids) is a must-do when prescribing any of the dietary approaches revised in this article.

## Figures and Tables

**Table 1:** Nutritional interventions in clinical practice guidelines for management of nonalcoholic fatty liver disease (NAFLD).

Area	Nutritional and Lifestyle Interventions	
	EASL-EASD-EASO [7]	AASLD-ACG-AGA [8,9]
Energy intake	↓ 500–1000 kcal/day	↓ 500–1000 kcal/day
Macronutrient composition	- Low-to-moderate fat and moderate-to-high carbohydrate intake - Low-carbohydrate ketogenic diets or high-protein - Macronutrient should be adjusted according to Mediterranean diet	- Not specified - ω-3 fatty acids may be considered to treat hypertriglyceridemia in patients with NAFLD
Fructose intake	Avoid fructose-containing beverages and foods	Not specified
Alcohol intake	Strictly keep alcohol below the daily risk threshold (30 g, men; 20 g, women)	- Patients with NAFLD should not consume heavy amounts of alcohol - There are insufficient data to make recommendations with regard to non-heavy consumption of alcohol by individuals with NAFLD
Coffee drinking	No liver-related limitations	Not specified
Micronutrients	Short-term treatment with vitamin E (800 IU/day) may be used in non-cirrhotic non-diabetic NASH cases	Vitamin E (800 IU/day) in non-diabetic NASH cases (not recommended to treat NASH in patients with diabetes, NAFLD without liver biopsy, NASH cirrhosis or cryptogenic cirrhosis)
Body weight loss recommendations	- ↓ 500–1000 g/week - 7–10% total weight loss target in overweight/obese NAFLD cases - Long-term maintenance approach, combining physical activity according to the principles of cognitive-behavioral treatment	- 3–5% total weight loss appears necessary to improve steatosis - 7–10% total weight loss is needed to improve histopathological features of NASH, including fibrosis - Weight loss is achieved by hypoenergetic diet alone or along with increased physical activity
Exercise/physical activity	- 150–200 min/week of moderate intensity aerobic physical activities in 3-5 sessions are generally preferred (brisk, walking, stationary cycling) - Resistance training has shown effectiveness and promotes musculoskeletal fitness, with effects on metabolic risk factors - High rates of inactivity-promoting fatigue and daytime sleepiness reduce compliance with exercise	- Moderate- intensity exercise - Exercise alone may reduce hepatic steatosis but its ability to improve other aspects of liver histology remains unknown

AASLD-ACG-AGA: American Association for the Study of Liver Diseases (AASLD), American College of Gastroenterology (ACG), American Gastroenterological Association (AGA); EASL-EASD-EASO: European Association for the Study of Liver (EASL), European Association for the Study of Diabetes (EASD), European Association for the Study of Obesity (EASO).

**Table 2:** Clinical trials comparing the efficacy of diverse dietary approaches in NAFLD.

Dietary Approaches	Study Design, Duration	N, Participant Features	Dietary Intervention, Macronutrient Distribution	Body Weight Outcomes	Changes in Liver Steatosis	Changes in Liver Enzymes (ALT/AST)	Reference
LFD vs. MedDiet vs. LCD	Randomized controlled, parallel, 2 years	322, adults with moderate obesity	- **LFD**: low-fat, restricted-energy diet with 1500/1800 kcal/day (30% fat with 10% saturated fat and 300 mg of cholesterol/day) - **MedDiet:** moderate-fat, restricted-energy Mediterranean diet with 1500/1800 kcal/day (35% fat with 30–45 g/day of olive oil and <20 g/day of nuts) - **LCD:** low-carbohydrate, non-restricted-energy diet (20 g CHO/day with a gradual increase to a maximum of 120 g/day)	Significantly weight loss in MedDiet and LCD groups: - LFD: −2.9 kg - MedDiet: −4.4 kg - LCD: −4.7 kg	NE	↓ ALT with MedDiet and LCD	Shai, I. 2008. Israel [25]
LFD vs. LCD	Randomized controlled, parallel, 6 months	170, adults with overweight and obesity	Energy-restricted diets for both groups (30% daily energy deficit, to a minimum of 1200 kcal/day): - **LFD:** <20% fat, 0.8 g/kg body weight protein, and remaining energy for CHO. - **LCD:** 30% fat, 0.8 g/kg body weight protein, <90 g/day CHO	Both diets similarly decreased body weight: - LFD: −6.5 kg - LCD: −7.5 kg	Comparable reduction of IHL% based on ^1^H-MRS: - LFD: −42% - LCD: −47%	- LFD: ↓ ALT, ↓ AST - LCD: ↓ AST	Haufe, S. 2011. Germany [26]
LFD vs. MedDiet	Single-blinded, randomized controlled, parallel, 12 weeks	56, adults with NAFLD (determined by MRS)	Both interventions were ad libitum isoenergetic diets: - **LFD:** 50% CHO, 30% fat, 20% protein - **MedDiet:** 40% CHO, 35–40% fat, 20% protein	Both diets similarly decreased body weight: - LFD: −1.6 kg - MedDiet: −2.1 kg	Comparable reduction of IHL% based on MRS: - LFD: −25% - MedDiet: −32.4%	↓ ALT in both groups	Properzi, C. 2018. Australia [27]
LFD vs. LCD	Randomized controlled, parallel, 8 weeks	32, children/adolescents with obesity and NAFLD (determined by ALT and/or US)	Family-based diet intervention. - **LFD:** based on the USDA MyPlate Daily Food Plan for teenagers (55% CHO, 20% fat, 25% protein) - **LCD:** designed to minimize refined CHO, high glycemic grains and fructose (<25% CHO, >50% fat, 25% protein)	Only the LCD group experienced significantly body weight loss: - LFD: −0.4% - LCD: −2.4%	Based on MRI, the IHL% did not differ with diet, but declined significantly only within LCD group (−32%)	Only in the LCD group: ↓ ALT ↓ AST	Goss, A. 2020. USA [28]
MedDiet/LCD vs. LFD	Randomized controlled, 18 months	278, adults with abdominal obesity/dyslipidemia	Diets aimed for moderate, long-term weight loss. - **MedDiet/LCD:** regimen combined the Mediterranean and low-carbohydrate diets (<40 g/day CHO with a gradual increase up to 70 g/day, an increased protein and fat intake according to the MedDiet, 28 g/day of walnuts) - **LFD:** 30% fat (with 10% saturated fat and 300 mg of cholesterol/day) and increased dietary fiber	In the entire cohort: moderate weight loss (−3.1%)	Based on MRI, the MedDiet/LCD group experienced greater %IHL reduction than LFD at 18 months: - MedDiet/LCD: −4.2% * - LFD: −3.8% * * absolute units	%IHL reduction was associated with ↓ ALT	Gepner, Y. 2019. Israel [29]
KD vs. low-energy diet	Semi-randomized, parallel, 2 weeks	18, adults with NAFLD (liver function tests, and 14/18 biopsy samples)	- **KD:** <20 g/day CHO - **Low-energy diet:** 1200/ 1500 kcal/day	Similar weight loss: - KD: −4.6 kg - Low-energy diet: −4.0 kg	Based on ^1^H-MRS, the KD resulted in significantly greater IHL% reduction: - KD: −55% - Low-energy diet: −28%	↓ AST in both groups, ALT did not change in both groups	Browning, J. 2011. USA [30]
DASH vs. low-energy diet	Randomized controlled, parallel, 8 weeks	60, adults with overweight/obesity and NAFLD (determined by US)	Both diets were energy restricted with 52–55% CHO, 30% fat, and 16–18% protein, however DASH diet was rich in fruits, vegetables, whole grains, and low-fat dairy products and low in saturated fats, cholesterol and refined grains	DASH diet resulted in significant weight loss: - DASH diet: −3.8 kg - -Low-energy diet: −2.3 kg	Both diets led a significant reduction in percentage of NAFLD grades, but greater percentage of patients in the DASH group had decreased grade of NAFLD	↓ ALT, significant greater reduction with DASH diet	Razavi Zade M, 2015. Iran [31]
MedDiet vs. low fat-high carbohydrate diet (LF/HCD)	Randomized, crossover, 6 weeks	12, adults with biopsy-proven NAFLD and without diabetes	All subjects undertook both diets with 6-week washout period in-between. - **MedDiet:** traditional Cretan MedDiet with 40% CHO, 40% fat, 20% protein - **LF/HCD:** 50% CHO, 30% fat, 20% protein	Slight and similar weight loss with both diets: - MedDiet: −1.0 kg - LF/HCD: −2.4 kg	Based on ^1^H-MRS, significant reduction in IHL% with MedDiet compared to control: - MedDiet: −39% - LF/HCD: −7%	ALT did not significant change with either diet	Ryan, M. 2013. Australia [32]
MedDiet + EVOO vs. MedDiet + Nuts vs. LFD	Multicenter randomized controlled, parallel, 6 year follow-up	276, adults with high cardiovascular risk (participants of PREDIMED-Malaga trial)	- **MedDiet:** traditional MedDiet supplemented with either EVOO (1 L/wk) or mixed nuts (30 g/day) - **LFD:** advice to reduced fat based on the National Cholesterol Education Program	In the MedDiet + Nuts group, BMI was 0.100 points lower per year compared to LFD	Based on Fatty Liver Index, dietary MedDiet could delay or slow down the progression of NAFLD	NE	Cueto-Galán R. 2017. Spain [33]
MedDiet + EVOO vs. MedDiet + Nuts vs. LFD	Multicenter, randomized controlled, parallel, 3 year follow-up	100, adults with high cardiovascular risk (Bellvitge-PREDIMED center)	- **MedDiet**: traditional MedDiet supplemented with either EVOO (1 L/wk) or mixed nuts (30 g/day) - **LFD:** advice to reduced fat	BMI did not change within groups, and was similar among groups	Based on MRI, at 3 y follow-up the IHL % was similar among groups (1.2%, 2.7% and 4.1% for MedDiet + EVOO vs. MedDiet + Nuts vs. LFD), and steatosis prevalence was lower in the MedDiet + EVOO group (8.8%, 33.3% and 33.3%, respectively)	At 3 y follow-up, similar ALT levels among groups	Pintó, X. 2019. Spain [34]
Low-glycemic index (GI) MedDiet vs. Control diet	Double-blind, randomized controlled, parallel, 6 months	98, adults with moderate or severe NAFLD (determined by US)	- **Low-GI MedDiet:** energy unrestricted diet, all foods had a low glycemic index, diet included olive oil and ω-3 fatty acids, 50% CHO, 30% fat (10% saturated fats) and 15–20% protein - **Control diet:** Based on INRAN guidelines	Not reported	Based on US, the low-GI MedDiet induced greater decrease in NAFLD score	↓ ALT in both groups	Misciagna, G. 2017. Italy [35]

ALT: alanine aminotransferase; AST: aspartate aminotransferase; BMI: body mass index; CHO: carbohydrates; EVOO: extra virgin olive oil; IHL: intrahepatic lipids; INRAN: Italian National Research Institute for Foods and Nutrition; KD: ketogenic diet; LCD: low-carbohydrate diet; LFD: low-fat diet; MedDiet: Mediterranean diet; MRI: magnetic resonance imaging; MRS: magnetic resonance spectroscopy; NE: not evaluated; US: ultrasound.

**Table 3:** Summary of non-comparative studies of diverse dietary approaches in NAFLD.

Study Design, Duration	N, Participant Features	Dietary Intervention, Macronutrient Distribution	Body Weight Outcomes	Changes in Liver Steatosis	Changes in Liver Enzymes (ALT/AST)	Reference
**Very low-carbohydrate diet (ketogenic diet)**						
Single-arm, 6 days	10, adults with overweight and obesity	Diet provided ~1440 kcal/day: ~6% CHO (≤25 g/day), ~64% fat and 28% protein	3% of weight loss	Based on ^1^H-MRS, IHL% decreased by ~31%	ALT did not change, AST/ALT ratio increased significantly by ~34%	Luukkonen, P. 2020. Finland [36]
Single-arm, 6 months	5, adults with obesity and NAFLD (determined by biopsy)	Diet provided <20 g/day CHO, with nutritional supplementation	Mean body weight change was −12.8 kg (0 to −25.9 kg)	Based on post-treatment biopsies, 4/5 presented histological improvements in steatosis as well as inflammatory grade and fibrosis	↓ ALT, but not statistically significant reductions	Tendler, D. 2007. USA [37]
Single-arm, 2 weeks (1st cohort)	1st cohort: 10 adults with overweight/obesity and NAFLD (determined by MRS)	Isoenergetic ketogenic diet with increased protein content that provided 3115 kcal/day and 23–30 g/day CHO (4% CHO, 72% fat and 24% protein); daily energy was increased when weight decreased by >0.2 kg between two study days	Minimal weight loss: −1.8%	Based on MRS, IHL% significantly decreased by 43.8%, the reduction was significant just 1 day after diet intervention	↓ AST	Mardinoglu, A. 2018. Sweden [38]
**Dietary approaches following the Mediterranean style diet pattern**						
Single-arm, 6 months	90, adults without diabetes, with overweight/obesity, and NAFLD (determined by US)	MedDiet and pro-active lifestyle intervention	Significant decrease of BMI (from the first month of intervention)	Based on US, (steatosis was graded 0–3 according to bright liver score, BLS): a significant decrease of BLS only after 6 months of intervention	ALT did not change	Trovato, FM. 2014. Italy [39]
Single-arm, 12 weeks	14, adults with overweight, metabolic syndrome and NAFLD (determined by US)	Spanish KD MedDiet: A protein KD, unlimited energy diet with < 30g/day CHO that included virgin olive oil, and ω-3 fatty acids from fish as main fat sources, moderate red wine intake, green vegetables and salads as main CHO sources, and fish as main protein source	Significant body weight loss (~14 kg)	Based on US, complete fatty liver regression in 21.4% of patients, and an overall reduction in 92.86%	↓ ALT ↓ AST	Pérez-Guisado, J. 2011. Spain [40]

ALT: alanine aminotransferase; AST: aspartate aminotransferase; BMI: body mass index; CHO: carbohydrates; IHL: intrahepatic lipids; KD: ketogenic diet; MedDiet: Mediterranean diet; MRS: magnetic resonance spectroscopy; US: ultrasound.

**Table 4:** Dietary approaches based on macronutrient restriction.

Diet	Macronutrient Limitation
Very low-fat diet	<20% of daily energy intake
Low-fat diet	<30% of daily energy intake
Very low-carbohydrate diet (ketogenic diet)	<20 to 50 g/day or <5 to 10% of daily energy intake
Low-carbohydrate diet	50 to 130 g/day or 10 to <40% of daily energy intake

## Abbreviations

ADF	alternate-day fasting
ALT	alanine aminotransferase
AST	aspartate aminotransferase
BMI	body mass index
CVD	cardiovascular disease
DASH	dietary approaches to stop hypertension
HOMA-IR	homeostatic model assessment of insulin resistance
IF	intermittent fasting
KD	ketogenic diet
LCD	low-carbohydrate diet
LFD	low-fat diet
MedDiet	Mediterranean diet
MUFA	monounsaturated fatty acids
NAFLD	nonalcoholic fatty liver disease
NASH	nonalcoholic steatohepatitis
SS	simple steatosis
T2DM	diabetes mellitus type 2
TRF	time-restricted feeding.
